# The Functional Neuroanatomy of Letter-Speech Sound Integration and Its Relation to Brain Abnormalities in Developmental Dyslexia

**DOI:** 10.3389/fnhum.2019.00021

**Published:** 2019-02-01

**Authors:** Fabio Richlan

**Affiliations:** Centre for Cognitive Neuroscience and Department of Psychology, University of Salzburg, Salzburg, Austria

**Keywords:** audiovisual integration, brain, development, dyslexia, grapheme-phoneme conversion, letter-speech sound integration, magnetic resonance imaging, reading

## Abstract

This mini-review provides a comparison of the brain systems associated with developmental dyslexia and the brain systems associated with letter-speech sound (LSS) integration. First, the findings on the functional neuroanatomy of LSS integration are summarized in order to obtain a comprehensive overview of the brain regions involved in this process. To this end, neurocognitive studies investigating LSS integration in both normal and abnormal reading development are taken into account. The neurobiological basis underlying LSS integration is consequently compared with existing neurocognitive models of functional and structural brain abnormalities in developmental dyslexia—focusing on superior temporal and occipito-temporal (OT) key regions. Ultimately, the commonalities and differences between the brain systems engaged by LSS integration and the brain systems identified with abnormalities in developmental dyslexia are investigated. This comparison will add to our understanding of the relation between LSS integration and normal and abnormal reading development.

## Developmental Dyslexia

Developmental dyslexia is a neurocognitive disorder characterized by a severe and persistent impairment in the acquisition of reading skills. According to the diagnostic criteria of DSM-IV (American Psychiatric Association, 2000) and ICD-10 (World Health Organization, 2007), performance in reading accuracy, fluency, comprehension and/or spelling is substantially below the performance expected from the person’s chronological age, intelligence, motivation, sensory acuity and educational environment. In addition, these difficulties significantly interfere with academic achievement or activities in everyday life requiring reading skills.

During the last two decades, there has been significant advance in the neurobiological understanding of developmental dyslexia. Across many languages and writing systems, studies using neurocognitive methods have identified brain regions critically involved in typical and dyslexic reading using functional magnetic resonance imaging (fMRI; e.g., Eden et al., 1996; Shaywitz et al., 1998; Temple et al., 2003; Siok et al., 2004; Gaab et al., 2007; Hoeft et al., 2007; van der Mark et al., 2009), electroencephalography (EEG; e.g., Duffy et al., 1980; Brandeis et al., 1994; Maurer et al., 2007), magnetoencephalography (MEG; e.g., Helenius et al., 1999; Simos et al., 2000; Salmelin, 2007), and positron-emission tomography (PET; e.g., Horwitz et al., 1998; Brunswick et al., 1999; Paulesu et al., 2001).

Qualitative narrative reviews and quantitative meta-analyses of neuroimaging studies have converged on a functional neuroanatomical model of developmental dyslexia. Specifically, altered brain activation in dyslexic readers was consistently reported in left posterior temporo-parietal (TP) cortex (middle and superior temporal, supramarginal and angular gyri), left occipito-temporal (OT) cortex (inferior temporal and fusiform gyri), and left frontal cortex (inferior frontal and precentral gyri). For the posterior brain regions (i.e., TP and OT cortices), the dominant finding is dyslexic underactivation compared with typical readers, while the picture is less clear for the anterior regions. Objective meta-analytic evidence speaks for dyslexic overactivation in the left precentral gyrus and underactivation in the left inferior frontal gyrus (IFG; Richlan et al., 2009, 2011; Martin et al., 2016; Hancock et al., 2017). In addition, there are occasional reports on other bilateral cortical, subcortical, and cerebellar dyslexic activation abnormalities but consistency across studies is scarce.

## Limitations and Open Issues

Importantly, dyslexic brain dysfunctions were predominantly assessed in the context of whole-word studies in the visual modality (i.e., studies visually presenting words or nonwords) utilizing reading-related tasks (e.g., lexical decision, semantic judgment, rhyme judgment, etc.). Undoubtedly, these studies have contributed tremendously to our understanding of the neural mechanisms during visual word recognition in typical and dyslexic readers (for a recent overview see Mascheretti et al., 2017). To what extent these findings generalize to natural reading processes, and especially to normal and abnormal reading development—requiring the initial integration of letters and speech sounds and the subsequent automation of this process—is an open issue.

Unfortunately, comparatively few studies investigated brain responses of dyslexic readers in relation to unimodal auditory stimulation (e.g., Corina et al., 2001; Gaab et al., 2007), and even fewer did so in relation to multimodal audiovisual stimulation (e.g., Blau et al., 2009; Kronschnabel et al., 2014). Multimodal audiovisual integration—particularly the binding of letters (or graphemes) and speech sounds (phonemes)—is a crucial process particularly during the early stages of literacy acquisition. Understanding of these proximal (neuro-) cognitive functions at the core of learning to read is an absolute necessity for a holistic understanding of typical and dyslexic reading development.

To this end, this mini-review summarizes the findings on the functional neuroanatomy of letter-speech sound (LSS) integration in order to obtain a comprehensive overview of the brain regions involved in this process. These brain regions are consequently compared with existing neurocognitive models of reading-related functional and structural brain abnormalities in developmental dyslexia. The investigation of the commonalities and differences between the brain systems engaged by LSS integration and the brain systems identified with abnormalities in developmental dyslexia will add to our understanding of the relation between letter-speech sound integration and normal and abnormal reading development.

## Letter-Speech Sound Integration

It has been aptly argued that the development of automated LSS integration plays a crucial role in the acquisition of fluent reading skills (e.g., Blomert, 2011). Consequently, failure to develop automated LSS integration results in an impairment of reading fluency. Therefore, a close link has been suggested between the development of automated processing of LSS associations and the emergence of a functional neuroanatomical system for skilled reading. Both behavioral and functional neuroimaging studies have evidenced less efficient LSS integration in children and adults with dyslexia compared with typically reading controls (e.g., Blau et al., 2009, 2010). In addition, recent intervention studies have demonstrated that training LSS correspondences could be a promising way to remediate slow and effortful reading in developmental dyslexia (e.g., Fraga González et al., 2015).

As explained by Blomert (2011), learning to read in alphabetic orthographies starts with learning a script code consisting of LSS pairs. Typically developing children learn the associations between letters (or graphemes) and speech sounds (phonemes) within months—often even before the onset of formal reading instruction. It takes, however, considerably longer to automatically process these LSS associations as newly constructed audiovisual (AV) objects. In beginning dyslexic readers—maybe as the result of an independent deficit or as a consequence of other deficits—this fundamental coupling of letters and speech sounds is substantially disturbed and the difficulties frequently persist into adulthood.

Blomert (2011) hypothesized that a specific deficit in the binding of sublexical orthographic and phonological information may not only constitute the immediate source of reading problems in developmental dyslexia, but may also explain the severe and persistent deficit regarding reading fluency—the lead symptom of dyslexia in shallow alphabetic orthographies (e.g., Wimmer, 1993; Torppa et al., 2010; Landerl et al., 2013). Undoubtedly, the proximal cause of developmental dyslexia is a highly controversial topic and the field certainly does not lack hypotheses about underlying (neuro-) cognitive deficits. The present mini-review is aimed at highlighting the possible role of an LSS integration deficit in dyslexia. In doing so, it does not deny or exclude other potentially relevant deficit explanations for the cause of developmental reading problems.

As explained in detail in the next section, in skilled readers LSS integration is linked to regions of the bilateral auditory cortex including the planum temporale (PT) and the bilateral heteromodal superior temporal sulcus (STS). The initial formation and subsequent automation of newly constructed grapheme-phoneme associations influences letter-specific processing and the build-up of visual-orthographic representations in the left ventral OT cortex. In developmental dyslexia, a neurocognitive deficit in the integration of letters and speech sounds is thought to impede the binding of orthographic and phonological information and, consequently, the emergence of the left ventral OT “reading skill zone” required for fast, fluent, and seemingly effortless reading.

Regarding the automation of LSS associations, important evidence comes from electrophysiology (i.e., EEG) studies (e.g., Froyen et al., 2008, 2009, 2011; Žarić et al., 2014, 2015). In these studies the mismatch negativity (MMN) is used, which is a valid indicator of automatic processing. For example, Froyen et al. (2009) showed that advanced readers (4 years of reading instruction) but not beginning readers (1 year of reading instruction) exhibited an enhanced MMN amplitude indicating fast and automatic LSS integration. Furthermore, Froyen et al. (2011) reported that in 11-year-old dyslexic children this response pattern was absent. Interestingly, although lacking the early, automatic processing stage, the dyslexic children showed a late negativity effect, which was similar to that of beginning readers and interpreted as reflecting non-automatic LSS matching.

## Functional Neuroimaging Studies on Letter-Speech Sound Integration

**Figure 1:**
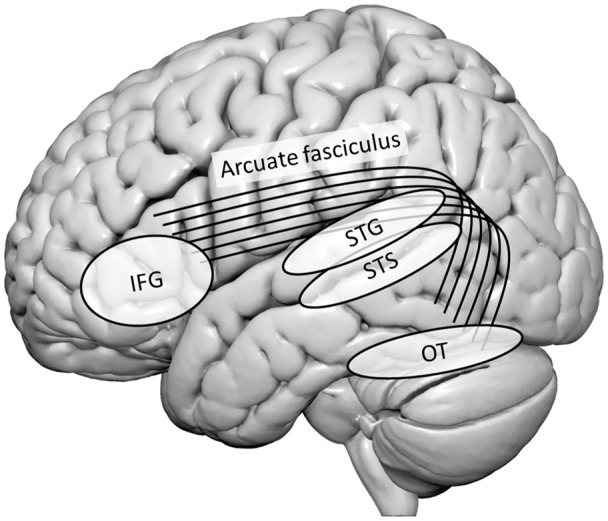
Schematic overview of the most important brain regions discussed in this mini-review and their interconnections *via* the arcuate fasciculus. IFG, inferior frontal gyrus; OT, occipito-temporal cortex; STG, superior temporal gyrus; STS, superior temporal sulcus.

Functional neuroimaging studies have identified several brain regions associated with LSS integration. These include bilateral temporal, OT, and inferior frontal regions. Specifically, a major role in basic sensory AV integration is attributed to the bilateral heteromodal STS and adjacent superior temporal gyrus (STG) and PT. More specifically, evidence for crucial engagement of the bilateral STS in grapheme-phoneme conversion was provided by the presence of congruency effects (i.e., differences between LSS pairs with congruent or incongruent orthographic and phonological information) in typical readers (e.g., van Atteveldt et al., 2004, 2007). Figure 1 provides a schematic overview of the most important brain regions discussed in this mini-review and their interconnections *via* the arcuate fasciculus.

In order to disentangle basic sensory aspects from higher-level associative (e.g., orthographic-phonological) aspects of AV integration, many of the functional neuroimaging studies on LSS integration use the following rationale (see Hocking and Price, 2008): activation in response to multisensory AV stimuli is compared with activation in response to unisensory auditory and unisensory visual stimuli to identify basic sensory aspects of AV integration. In some cases the multisensory AV stimulation results in higher activation compared with the summed unisensory auditory + visual stimulation (i.e., super-additivity effect), whereas in other cases the multisensory AV stimulation results in lower activation compared with the summed unisensory auditory + visual stimulation (i.e., sub-additivity effect). Both effects can be interpreted as indicating aspects of basic sensory AV integration. There are, however, limitations to this approach due to potential blood-oxygen-level-dependent (BOLD) saturation effects in fMRI (Goebel and van Atteveldt, 2009).

In order to test for higher-level associative (e.g., orthographic-phonological) aspects of AV integration, activation in response to congruent LSS pairs is compared with activation in response to incongruent LSS pairs (i.e., congruency effect). Congruent means that the orthographic information represented by the visual letter stimulus matches the phonological information represented by the (simultaneously or sequentially presented) auditory speech sound stimulus. Accordingly, in incongruent LSS pairs this information does not match. Usually, the presence of a congruency effect (regardless of whether congruent LSS pairs result in higher activation compared with incongruent LSS pairs or vice versa) is taken as indicator for the engagement of a certain brain region in AV grapheme-phoneme conversion.

The tasks employed by the different functional neuroimaging studies vary considerably and—unsurprisingly—were shown to have a substantial effect on the degree of activation of the identified brain regions (van Atteveldt et al., 2007) and on the presence and/or direction of the congruency effect (Kronschnabel et al., 2014). The tasks employed include passive perception (viewing and/or listening; e.g., van Atteveldt et al., 2004), active matching (i.e., indicating *via* button press whether the letter and the speech sound match; e.g., van Atteveldt et al., 2007), specific speech sound target detection (i.e., detecting /a/; e.g., Blau et al., 2008), non-letter and non-speech sound target detection (i.e., detecting simple visual – ### –, auditory—piano sound—and AV targets among LSS pairs; e.g., Kronschnabel et al., 2014) and one-back task (i.e., detecting repeated stimuli; e.g., Francisco et al., 2018).

Across studies and despite different functional activation tasks, age groups and orthographies, the most consistently identified brain region associated with both basic sensory and higher-level associative AV integration seems to be the bilateral heteromodal STS. Here the typical findings are: (i) higher activation for multisensory compared with unisensory stimulation; and (ii) higher activation for congruent compared with incongruent LSS pairs in skilled readers (e.g., van Atteveldt et al., 2004). As already mentioned, the exact locations and response profiles of the activated brain regions depend on the in-scanner functional activation task. In addition, the response might be blurred by temporal limitations of the BOLD fMRI signal. In this case, EEG or MEG studies (e.g., Herdman et al., 2006; Froyen et al., 2011; Žarić et al., 2015) providing high temporal resolution might be more informative.

Another method to circumvent specific limitations of the BOLD signal, namely saturation effects and spatial averaging, is by using an fMRI adaptation design. In this design, the well-known phenomenon of repetition suppression (i.e., the reduced neural activity in response to stimulus repetitions) is utilized in order to investigate the functional specificity of the neural populations within voxels. van Atteveldt et al. (2010) used such a design and identified several small clusters along the STG and STS showing stronger adaptation in response to repetitions of congruent compared with incongruent LSS pairs. This finding was taken as evidence for the existence of multisensory neurons in the STG/STS that are tuned to AV content relatedness.

In addition to the specific adaptation effect in the STG and STS, van Atteveldt et al. (2010) identified a network of bilateral OT regions that showed a more general adaptation effect. That is, these regions adapted to repetitions of both congruent and incongruent LSS pairs, indicating sensitivity to letters, speech sounds or both. Activation in other regions often identified in fMRI studies on LSS integration, like the IFG, was assumed to be more related to the type of task employed and corresponding explicit decision making in active matching paradigms (Blomert, 2011). Likewise, activation in the inferior parietal lobule (IPL) is often related to task demands requiring executive functions, particularly in the presence of ambiguity (Oberhuber et al., 2016; Vignali et al., 2019).

Based on the results from a carefully designed fMRI study, Hocking and Price (2008) postulated a more general role of the bilateral posterior STS in conceptual matching, not necessarily restricted to AV integration. Most importantly, they found that the bilateral posterior STS responds in the same way to crossmodal AV conceptual matching as to intramodal auditory or intramodal visual matching when task, attention and stimuli are controlled. They concluded that the posterior STS is not specifically dedicated to multimodal integration but is part of a bilateral brain network including OT, IFG and IPL regions subserving conceptual matching, irrespective of input modalities.

In line with the idea of a functional brain network supporting AV, auditory-auditory or visual-visual conceptual matching, Blomert (2011) emphasized the importance of the gradual tuning of OT and IPL regions for increasingly automated LSS integration. This tuning and automation constitutes one of the first milestones in reading acquisition and provides the basis for the emergence of an efficient functional neuroanatomical network for the integration of letters and speech-sounds (van Atteveldt et al., 2009) and for skilled reading (Brem et al., 2010; Schurz et al., 2014a; Martin et al., 2015; Schuster et al., 2015). Exactly this functional neuroanatomical network was shown to be disrupted in developmental dyslexia (e.g., Richlan, 2012), as will be discussed in detail in the next section.

## The Functional Neuroanatomy of Letter-Speech Sound Integration and Its Relation to Brain Abnormalities in Developmental Dyslexia

In the field of developmental dyslexia functional neuroimaging studies on LSS integration are relatively new (Blau et al., 2009, 2010; Holloway et al., 2013; Kronschnabel et al., 2014; Karipidis et al., 2017, 2018). Blau et al. (2009, 2010) followed up on the seminal fMRI studies by van Atteveldt et al. (2004, 2007) and used their AV LSS integration paradigm with dyslexic adults (Blau et al., 2009) and with dyslexic children (Blau et al., 2010). Basically, the dyslexic readers did not exhibit the behavioral and neurofunctional congruency effects demonstrated by the typical readers. That is, the dyslexic readers did not show higher activation for congruent compared with incongruent LSS pairs in the brain regions (e.g., STS) identified as being part of the AV integration network in skilled readers (see e.g., van Atteveldt et al., 2004).

Furthermore, strong evidence for structural abnormalities (i.e., less gray matter volume) in STG and STS regions in developmental dyslexia was reported in quantitative coordinate-based meta-analyses and multi-center studies across different laboratories and countries (Richlan et al., 2013; Eckert et al., 2016). Taken together, these findings were interpreted as indicating a disruption in the functional neuroanatomical network supporting automated AV integration and grapheme-phoneme conversion in developmental dyslexia. Interestingly, two structural MRI studies with pre-reading children found that children with a family-risk for developmental dyslexia exhibited reduced gray matter volume in bilateral STG/STS regions even before formal reading instruction (Raschle et al., 2011; Black et al., 2012). Importantly, for these young children the reduction in gray matter volume can hardly be attributed to a reduced amount of reading experience.

Similar to the findings of the Dutch readers of Blau et al. (2009, 2010), Kronschnabel et al. (2014) reported activation differences between typical and dyslexic readers in congruency effects in a sample of native German-speaking Swiss adolescents. Brain regions identified with group differences included the STS, OT, IFG and IPL. Interestingly, the directionality of the congruency effect was different from the previous studies. This is most probably attributable to subtle differences in the experimental task—avoiding active monitoring of congruency condition by guiding the participants’ attention away from the LSS pairs (see previous section on tasks), orthographic depth of the investigated language (see Holloway et al., 2013 for similar results in native English readers) and/or developmental factors.

Recently, Karipidis et al. (2017, 2018) investigated the emergence of AV integration in pre-reading children at varying risk for developmental dyslexia by training artificial LSS correspondences. The artificial LSS pairs were familiarized in a single training session of about 10–30 min and consisted of unfamiliar false font characters coupled with familiar phonemes. The fMRI data acquired after the training session revealed associations between individual learning rate, phonological awareness and familial history of developmental dyslexia with degree of activation in a brain network consisting of bilateral STS/STG, OT, frontal and parietal regions.

The results of these functional neuroimaging studies are fully compatible with the notion of a gradual tuning of a distributed brain network subserving increasingly automated AV binding postulated by Blomert (2011). The specific crossmodal binding deficit between letters and speech sounds in impaired readers is thought to be reflected in defective functional and structural connectivity between the brain regions constituting the reading network in skilled readers including occipital, temporal, parietal and frontal brain regions (Richlan, 2012, 2014). The disrupted connectivity between uni- and multisensory brain regions particularly in temporal and occipital cortices may hamper the incremental emergence of fast and efficient single- and multi-letter recognition in the putative “reading skill zone” of the left ventral OT cortex in developmental dyslexia.

The left ventral OT cortex was identified as exhibiting underactivation in dyslexic readers compared with age-matched controls across experimental tasks (Richlan et al., 2009), age groups (Richlan et al., 2011) and orthographies (Paulesu et al., 2001; Martin et al., 2016). It was proposed that in typical readers the left ventral OT cortex is not only engaged by fast and effortless visual word processing but even more so by unfamiliar letter-string processing relying on phonological decoding (Richlan et al., 2010; Schurz et al., 2010; Wimmer et al., 2010). Therefore, the left ventral OT cortex in skilled readers serves as an interface area providing access from visual-orthographic information to phonological information (Price and Devlin, 2011).

In typical readers, left ventral OT, temporal and frontal regions are functionally connected, whereas in dyslexic readers this functional coupling is impaired. The reduced functional connectivity between left ventral OT and superior temporal/inferior frontal brain regions was shown for both reading-related (e.g., van der Mark et al., 2011; Olulade et al., 2015) as well as resting-state activation (e.g., Schurz et al., 2014b). Consistent with these observations are findings from neuroimaging studies on structural connectivity using diffusion tensor imaging. As evidenced by the meta-analysis by Vandermosten et al. (2012), dyslexic readers exhibit reduced integrity of the major white matter fiber tracts connecting the brain regions engaged during reading processes. Importantly, the main difference in structural integrity between typical and dyslexic readers was identified in the left TP white matter.

Although it is not entirely resolved which of various potential fiber tracts is specifically affected (see Ben-Shachar et al., 2007), convincing evidence points to the left arcuate fasciculus (Dehaene et al., 2015). It connects occipital, temporal, parietal, and frontal language regions and was shown to be among the first brain systems to anatomically change during reading acquisition. Specifically, an increase in fractional anisotropy and a decrease in perpendicular diffusivity indicated a microstructural improvement of the TP aspect of the arcuate fasciculus in response to learning to read (Thiebaut de Schotten et al., 2012; Yeatman et al., 2012). Based on these properties, the left arcuate fasciculus is assumed to play an important role particularly during early stages of reading development by subserving LSS integration and grapheme-phoneme conversion, which, in turn, constitutes the prerequisite for self-reliant phonological word decoding.

The idea that developmental dyslexia results from impaired connections between brain regions for vision and language was first put forward by Geschwind (1965a,b). Since—at least for shallow alphabetic orthographies—the dyslexic reading speed impairment was sufficiently explained by a reformulation of the phonological deficit explanation postulating an inefficient access from letters to otherwise intact phonemic information (Wimmer, 1993), this idea received new support (see Ramus and Szenkovits, 2008; Boets et al., 2013). As evidenced by modern-day neuroimaging, the visual-verbal speed deficit of dyslexic readers can be aptly attributed to functional and structural impairments in the TP and OT brain systems linking both lexical and sub-lexical orthographic and phonological information.

## Conclusions

According to the here presented literature, the development of automated LSS integration is thought to play a crucial role in the acquisition of fluent reading skills and disturbance of this development was shown to result in an impairment of reading fluency—the lead symptom of dyslexia in shallow alphabetic orthographies. Both behavioral and functional neuroimaging studies have evidenced less efficient LSS integration in children and adults with developmental dyslexia compared with typically reading controls—although certainly more research on the potential causal role of LSS integration deficits in developmental dyslexia is needed.

In skilled readers successful LSS integration is linked to regions of the bilateral auditory cortex including the PT and the bilateral heteromodal STS. The initial formation and subsequent automation of newly learned AV grapheme-phoneme associations influences letter-specific processing and the build-up of visual-orthographic representations in the left ventral OT cortex. In developmental dyslexia, a putative specific neurocognitive deficit in the crossmodal integration of letters and speech sounds is thought to impede the binding of orthographic and phonological information and, consequently, the emergence of the functional neuroanatomical brain system including the left ventral OT “reading skill zone,” the heteromodal TP cortex and frontal brain regions required for fast, fluent, and seemingly effortless reading.

## Author Contributions

FR conceived and wrote the manuscript.

## Conflict of Interest Statement

The author declares that the research was conducted in the absence of any commercial or financial relationships that could be construed as a potential conflict of interest.
